# Prognostic Significance of Preoperative Neutrophil-to-Lymphocyte Ratio in Patients With Meningiomas

**DOI:** 10.3389/fonc.2020.592470

**Published:** 2020-11-24

**Authors:** Yuki Kuranari, Ryota Tamura, Noboru Tsuda, Kenzo Kosugi, Yukina Morimoto, Kazunari Yoshida, Masahiro Toda

**Affiliations:** ^1^ Department of Neurosurgery, Keio University School of Medicine, Tokyo, Japan; ^2^ Department of Pathology, Keio University School of Medicine, Tokyo, Japan

**Keywords:** meningioma, neutrophil-to-lymphocyte ratio, World Health Organization grade I, recurrence, progression-free survival

## Abstract

**Background:**

Meningiomas are the most common benign intracranial tumors. However, even WHO grade I meningiomas occasionally show local tumor recurrence. Prognostic factors for meningiomas have not been fully established. Neutrophil-to-lymphocyte ratio (NLR) has been reported as a prognostic factor for several solid tumors. The prognostic value of NLR in meningiomas has been analyzed in few studies.

**Materials and Methods:**

This retrospective study included 160 patients who underwent surgery for meningiomas between October 2010 and September 2017. We analyzed the associations between patients’ clinical data (sex, age, primary/recurrent, WHO grade, extent of removal, tumor location, peritumoral brain edema, and preoperative laboratory data) and clinical outcomes, including recurrence and progression-free survival (PFS).

**Results:**

Forty-four meningiomas recurred within the follow-up period of 3.8 years. WHO grade II, III, subtotal removal, history of recurrence, Ki-67 labeling index ≥3.0, and preoperative NLR value ≥2.6 were significantly associated with shorter PFS (*P* < 0.001, < 0.001, 0.002, < 0.001, and 0.015, respectively). Furthermore, NLR ≥ 2.6 was also significantly associated with shorter PFS in a subgroup analysis of WHO grade I meningiomas (*P* = 0.003). In univariate and multivariate analyses, NLR ≥2.6 remained as a significant predictive factor for shorter PFS in patients with meningioma (*P* = 0.014).

**Conclusions:**

NLR may be a cost-effective and novel preoperatively usable biomarker in patients with meningiomas.

## Introduction

Meningioma is the most common primary brain tumor, accounting for 37.6% of all brain tumors (1). Approximately 80% of meningiomas are classified as WHO grade I (1). However, even benign WHO grade I meningiomas occasionally show rapid growth and may recur after total removal (2). The biological characteristics of meningioma have not been fully elucidated. The identification of prognostic biomarkers is warranted to optimize the treatment strategies.

To date, various prognostic factors for meningiomas have been described in previous studies, and among those factors, the most reliable clinical factors have been WHO grade and the extent of removal (EOR) (3, 4). Ki-67 labeling index (Ki-67 LI), which is frequently used to predict the prognosis of malignant tumors (5), has been reported to be useful in predicting meningioma recurrence (6, 7). However, other studies have not shown a significant correlation between Ki-67 LI and poor prognosis (8, 9). These factors are based on postoperative information; however, no preoperative prognostic factors have been established.

Recently, hematological inflammatory markers, such as neutrophil-to-lymphocyte ratio (NLR), lymphocyte-to-monocyte ratio (LMR), and platelet-to-lymphocyte ratio (PLR) have been reported to be poor prognostic indicators for various solid tumors (10–12). However, the significance of NLR in patients with meningiomas has not been analyzed extensively (13, 14). Furthermore, the association between peripheral and intratumoral inflammatory markers has not been analyzed in meningiomas ever.

Here, we investigated the prognostic significance of hematological inflammatory markers, including NLR, LMR, and PLR, in patients with meningiomas, and discussed the role of the inflammatory response in the tumor microenvironment.

## Materials and Methods

### Study Population and Clinical Data

We retrospectively analyzed data from patients who underwent surgery for meningiomas (WHO grade I–III) at our institute between October 2010 and September 2017. This study was approved by the Institutional Review Board (Reference number: 20050002), and written consent was obtained from all patients.

The exclusion criteria of this study were as follows: 1) patient aged < 18 years (n = 1); 2) patients who received steroids before preoperative laboratory test (n = 12); 3) patients with incomplete medical records (n = 20); 4) patients with neurofibromatosis type 2 (n = 2); 5) patients with a known history of whole-brain radiation therapy before surgery (n =2).

Clinical data including age at surgery, sex, primary/recurrent, WHO grade, EOR, tumor location, and peritumoral brain edema (PTBE) were obtained from hospital and electronic medical charts. The Simpson grading scale was used to evaluate the EOR (15). The EOR was categorized as gross total removal (GTR) (Simpson grade I–III) or subtotal removal (STR) (Simpson grade IV and V), as described previously (16). Surgical data were retrieved from operative reports and the removal rate was validated with routine postoperative head CT at 7 days after the operation. Gadolinium-enhanced T1-weighted MRI was used to evaluate the tumor location. Skull base location was defined as described previously (17). PTBE was evaluated on preoperative T2-weighted images or fluid-attenuated inversion recovery images (18).

Postoperative MRI was performed every 6–12 months. Tumor recurrence was defined as follows: 1) for patients with GTR, the appearance of new lesions at the prior surgical site and 2) for patients with STR, residual tumor growth (> 2 mm/year) (19).

Progression-free survival (PFS) was calculated from the date of surgery to the date of either tumor recurrence or death from meningioma. For patients with no confirmed recurrence, PFS was calculated from the date of surgery to the date of the last follow-up MRI.

### Laboratory Data

Routine preoperative laboratory test data were used for analysis. The absolute neutrophil, lymphocyte, monocyte, and platelet counts were collected. Subsequently, we calculated the following parameters; NLR (absolute neutrophil count divided by absolute lymphocyte count) (10), LMR (absolute lymphocyte count divided by absolute monocyte count) (13, 20), and PLR (absolute platelet count divided by absolute lymphocyte count) (13). Neutrophilia was defined as the absolute neutrophil count ≥ 7.5 x 10^9^/L, and lymphocytopenia as the absolute lymphocyte count < 1.5 x 10^9^/L, as reported previously (21).

### Histopathological Analysis

For histopathological analysis, we used paired (primary and recurrent) samples (26 tumors) obtained from 13 patients. None of the 13 patients had received chemotherapy or radiation therapy before tumor recurrence. The intratumoral neutrophils were assessed with their characteristic morphology using hematoxylin and eosin staining. Immunohistochemical staining was performed on 4-μm-thick sections of formalin-fixed paraffin-embedded tissues. The following steps were performed as described previously (22, 23). The primary antibodies were anti-Ki-67 antibody (1:200, M7240, Agilent DAKO, Santa Clara, CA, USA), anti-CD4 antibody (1:250, 1F6, Nichirei Biosciences, Inc., Tokyo, Japan), anti-CD8 antibody (1:200, ab17147, Abcam, Cambridge, UK), and anti-CD163 antibody (1:500, ab87099, Abcam). The primary antibodies were detected using the appropriate secondary antibodies (ImmPRESS Detection Systems, Vectorlabs, Burlingame, CA, USA). Diaminobenzidine was used for color development, and the products were visualized and photographed under a light microscope (Biorevo BZ-9000, Keyence Corporation, Osaka, Japan).

Immunohistochemical expression was assessed by two neurosurgeons and one neuropathologist who were blind to clinical information. Ki-67 LI was recorded as the percentage of tumor cells with positive nuclear staining at ×20 magnification. The cell counts were performed in regions of maximum immunoreactivity. For the assessment of neutrophils, and CD4, CD8, and CD163 + cells, the stained tissue sections were screened at ×4 magnification, and five hot spots were selected, as described previously (22, 23). The cells were counted manually at ×40 magnification. The mean numbers of neutrophils and positive cells per field were calculated.

### Statistical Analyses

We used GraphPad Prism 8 (GraphPad Software, San Diego, CA, USA) to perform statistical analyses. Receiver operating characteristic (ROC) curves were constructed to determine the optimal cut-off values of NLR, LMR, and PLR (based on Youden’s index) to predict the recurrence of meningioma after surgery. Subsequently, each variable was analyzed as a dichotomous variable, according to the optimal cut-off value. Ki-67 LI was dichotomized at 3.0, as reported previously (6, 24). Continuous variables were expressed as means±standard deviations. The chi-squared test was used to compare categorical variables, and the Mann-Whitney U test was used to compare continuous variables. PFS was estimated using the Kaplan-Meier method and log-rank analysis was used to compare survival curves between different subgroups. Cox’s proportional hazards method was used to investigate the influence of variables on PFS in univariate and multivariate analyses. A P value of < 0.05 was considered statistically significant.

## Results

### Patient Characteristics

A total of 160 patients (39 male and 121 female) with complete preoperative laboratory data available were included in this study (**Table 1**). The median follow-up period was 3.8 years (range: 0–8.9 years). The median age at operation was 61 years (range: 28–84 years). Twenty-seven patients (16.9%) were recurrent cases. There were 144 WHO grade I (90.0%), 14 grade II (8.8%), and two grade III (1.2%) meningiomas. GTR was achieved in 117 cases (73.1%). The histological subtypes of meningiomas are shown in **Supplementary Table 1**. One hundred and eight patients (67.5%) had skull base meningiomas; the other 52 (32.5%) had non-skull base meningiomas. PTBE was observed in 72 patients (45.0%). Forty-four meningiomas recurred within the follow-up period [32 WHO grade I (22.2%), 10 grade II (71.4%), and two grade III (100%) meningiomas]. Among WHO grade I meningiomas, meningothelial and transitional subtypes exhibited a higher frequency of recurrence (25.4% and 28.1%, respectively).

**Table 1:** Baseline characteristics (N = 160).

Clinical feature	All cases	Baseline NLR		*P*-value
		< 2.6	≥ 2.6	
		No. (%)	No. (%)	
Patient number	160	124 (77.5)	36 (22.5)	
Age (means±SD)		60.1±12.2	57.1±14.3	0.26
Sex				0.92
Male	39	30 (76.9)	9 (23.1)	
Female	121	94 (77.7)	27 (22.3)	
Tumor status				0.97
Primary	133	103 (77.4)	30 (22.6)	
Recurrent	27	21 (77.8)	6 (22.2)	
WHO grade				0.70
Grade I	144	111 (77.1)	33 (22.9)	
Grade II and III	16	13 (81.3)	3 (18.7)	
Extent of removal				0.47
Gross total removal	117	89 (76.1)	28 (23.9)	
Subtotal removal	43	35 (81.4)	8 (18.6)	
Location				0.013
Skull base	108	90 (83.3)	18 (16.7)	
Non-skull base	52	34 (65.4)	18 (34.6)	
Peritumoral brain edema (PTBE)				0.068
With PTBE	72	51 (70.8)	21 (29.2)	
Without PTBE	88	73 (83.0)	15 (17.0)	

NLR, neutrophil-to-lymphocyte ratio; SD, standard deviation; WHO, World Health Organization; PTBE, peritumoral brain edema.

### Laboratory Data

Preoperative laboratory data are shown in **Table 2**. Preoperative neutrophilia was observed in only one patient. Preoperative lymphopenia was observed in 48 patients (30%). The ROC curve showed NLR cut-off value of 2.6 as a predictive marker of tumor recurrence, with a sensitivity 34.1% and specificity 81.9%. The area under the curve was 0.55 (**Supplementary Figure 1**). Similarly, the optimal cut-off values for LMR and PLR were 5.3, and 140, respectively (**Supplementary Figure 1**). As shown in **Table 1**, the preoperative NLR did not differ with regard to age, sex, WHO grade, EOR, and PTBE. NLR ≥ 2.6 were significantly more common for non-skull base meningiomas than for skull base meningiomas.

**Table 2:** Preoperative laboratory data and hematological inflammatory markers.

Marker	Mean±SD
Neutrophil count (×10^9^/L)	3.53±1.26
Lymphocyte count (×10^9^/L)	1.78±0.55
NLR (Neutrophil-to-lymphocyte ratio)	2.14±1.03
LMR (Lymphocyte-to-monocyte ratio)	6.33±2.29
PLR (Platelet-to-lymphocyte ratio)	148.38±61.59

SD, standard deviation.

### Kaplan-Meier Analysis

**Figure 1:**
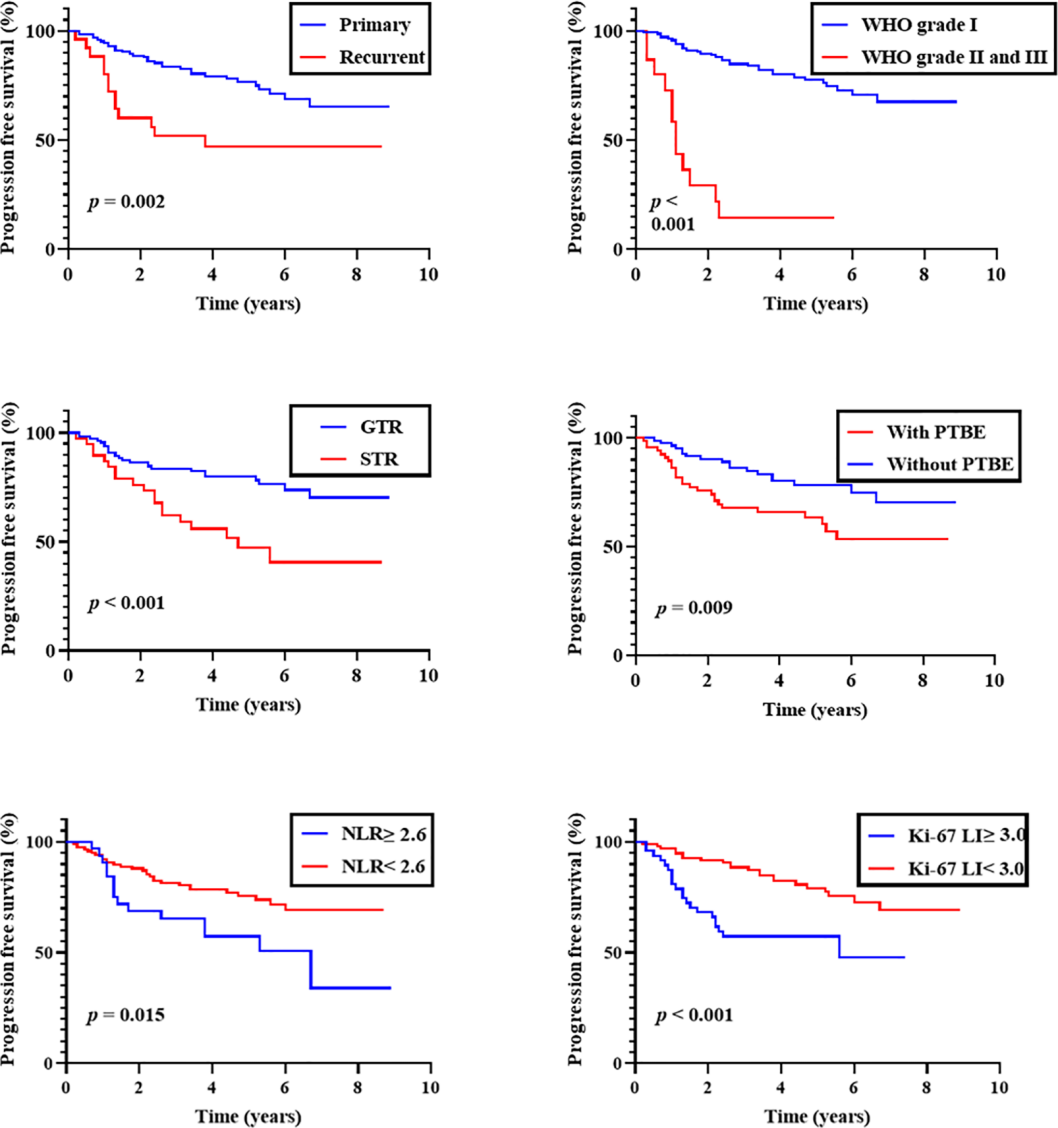
Progression-free survival of overall patients. Kaplan-Meier curves of progression-free survival stratified by primary/recurrent, WHO grade, the extent of removal, peritumoral brain edema (PTBE), preoperative neutrophil-to-lymphocyte ratio (NLR; cut-off 2.6), and Ki-67 labeling index (Ki-67 LI; cut-off 3.0).

Kaplan-Meier analysis showed that both WHO grade and EOR were correlated with shorter PFS (P < 0.001 for each). A history of recurrence and PTBE were also correlated with shorter PFS (P = 0.002 and 0.009, respectively). When we dichotomized NLR and Ki-67 LI at 2.6 and 3.0, both were predictive of shorter PFS (P = 0.015 and < 0.001, respectively; **Figure 1**). No other clinical factors were associated with shorter PFS.

### Univariate and Multivariate Analyses

To investigate the influence of variables on PFS, we performed univariate analysis with Cox’s proportional hazards model for age (≥ 60 versus < 60 years), sex, primary/recurrent, WHO grade (I versus II and III), EOR (GTR versus STR), tumor location (skull base versus non-skull base), PTBE (with PTBE versus without PTBE), absolute neutrophil count (continuous variable), lymphocytopenia (present versus not present), NLR (≥ 2.6 versus < 2.6), LMR (≤ 5.3 versus > 5.3), PLR (≥ 140 versus < 140), and Ki-67 LI (≥ 3.0 versus < 3.0; **Table 3**). Among these variables, a history of recurrence, WHO grade (II and III), EOR (STR), PTBE (with PTBE), NLR (≥ 2.6), LMR (≤ 5.3), and Ki-67 LI (≥ 3.0) were associated with shorter PFS and were included in the subsequent multivariate analysis [history of recurrence, hazards ratio (HR) = 2.64, 95% confidence interval (CI) = 1.38–5.06, P = 0.003; WHO grade, HR = 8.87, 95% CI = 4.42–17.80, P < 0.001; EOR, HR = 2.69, 95% CI = 1.48–4.91, P = 0.001; PTBE, HR = 2.18, 95% CI = 1.19–3.98, P = 0.011; NLR, HR = 2.13, 95% CI = 1.14–3.98, P = 0.018; LMR, HR = 2.39, 95% CI = 1.32–4.32, P = 0.004; Ki-67 LI, HR = 2.84, 95% CI = 1.55–5.21, P < 0.001].

**Table 3:** Univariate and multivariate analysis.

Variables	Univariate				Multivariate					
	*P*-value	HR	95%CI		*P*-value		HR		95%CI	
Age (≥ 60)	0.55	0.83	0.46–1.51							
Sex (Male)	0.097	1.73	0.91–3.31							
Tumor status (Recurrent)	**0.003**	**2.64**	**1.38**–**5.06**		**0.045**		**2.07**		**1.02**–**4.23**	
WHO grade (Grade II and III)	**<0.001**	**8.87**	**4.42**–**17.80**		**<0.001**		**10.01**		**3.71**–**27.03**	
Removal rate (Subtotal removal)	**0.001**	**2.69**	**1.48**–**4.91**		**<0.001**		**4.44**		**2.27**–**8.67**	
Location (Non-skull base)	0.21	1.47	0.80–2.71							
Peritumoral brain edema (With PTBE)	**0.011**	**2.18**	**1.19**–**3.98**		0.55		1.23		0.63–2.42	
Preoperative Neutrophil count*	0.20	1.00	0.9999–1.0003							
Preoperative Lymphocytopenia	0.16	1.54	0.84–2.82							
Preoperative NLR (≥ 2.6)	**0.018**	**2.13**	**1.14**–**3.98**		**0.022**		**2.29**		**1.13**–**4.64**	
Preoperative LMR (≤ 5.3)	**0.004**	**2.39**	**1.32**–**4.32**		0.10		1.74		0.89–3.38	
Preoperative PLR (≥ 140)	0.060	1.79	0.97–3.28							
Ki-67 LI (≥ 3.0)	**<0.001**	2.84	1.55–5.21		0.27		1.55		0.71–3.37	

HR, hazard ratio; CI, confidence interval; WHO, World Health Organization; PTBE, peritumoral brain edema; NLR, neutrophil-to-lymphocyte ratio; LMR, lymphocyte-to-monocyte ratio; PLR, platelet-to-lymphocyte ratio; Ki-67 LI, Ki-67 labeling index.

Bold font indicates statistical significance (P < 0.05).

*Continuous variable.

Multivariate analysis showed that a history of recurrence, WHO grade (II and III), EOR (STR), and NLR (≥ 2.6) were independent predictors of poor prognosis (history of recurrence, HR = 2.07, 95% CI = 1.02–4.23, P = 0.045; WHO grade, HR = 10.01, 95% CI = 3.71–27.03, P < 0.001; EOR, HR = 4.44, 95% CI = 2.27–8.67, P < 0.001; NLR, HR = 2.29, 95% CI = 1.13–4.64, P = 0.022) (**Table 3**).

### Subgroup Analysis

**Figure 2:**
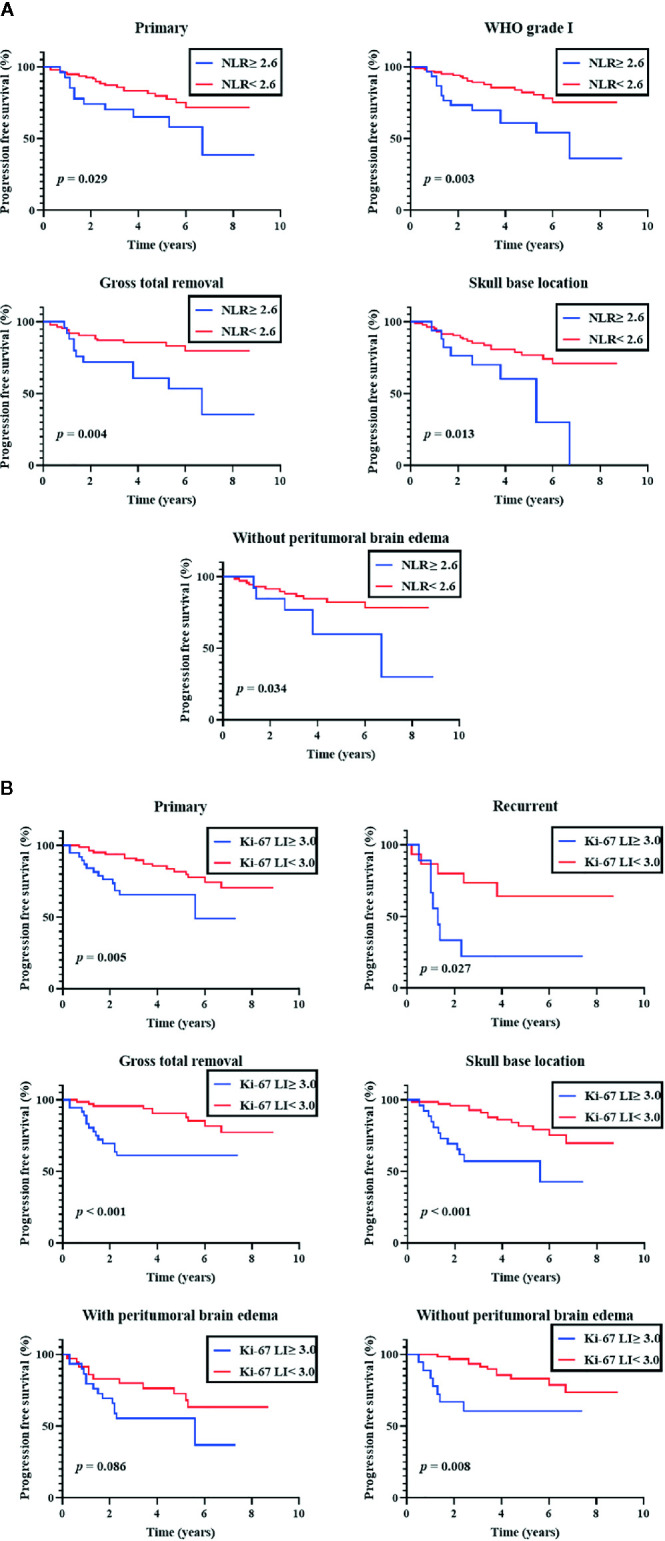
Subgroup analyses of progression-free survival. Kaplan-Meier curves of progression-free survival. **(A)** Subgroup analysis of preoperative neutrophil-tolymphocyte ratio (NLR; cut-off 2.6) **(B)** Subgroup analysis of Ki-67 labeling index (Ki-67 LI; cut-off 3.0).

An additional subgroup analysis was performed after stratifying cases by the primary/recurrent, WHO grade (I, II, and III), EOR (GTR and STR), tumor location (skull base and non-skull base), and PTBE (with and without PTBE) (**Figure 2**). In a subgroup of primary meningiomas, EOR (GTR), tumor location (SB), and PTBE (without PTBE), both preoperative NLR (≥ 2.6) and Ki-67 LI were significantly associated with shorter PFS (NLR, P = 0.029, 0.004, 0.013, and 0.034, respectively; Ki-67 LI, P = 0.005, < 0.001, < 0.001, and 0.008, respectively). However, in a subgroup of WHO grade I meningiomas, only preoperative NLR (≥ 2.6) was significantly associated with shorter PFS (NLR, P = 0.003; Ki-67 LI, P = 0.17). In a subgroup of recurrent meningiomas, NLR (≥ 2.6) was not significantly associated with shorter PFS (P = 0.32). In each subgroup, ROC curves were constructed to determine the optimal NLR cut-off value to predict recurrence with sensitivity and specificity (**Supplementary Table 2**).

### Immunohistochemical Analyses

To evaluate the association between peripheral NLR and intratumoral inflammatory markers, we analyzed neutrophils and CD8, CD4, and CD163+ cells from paired primary and recurrent tumor specimens. Elevated peripheral NLR was not correlated with the number of intratumoral neutrophils or CD8, CD4, or CD163+ cells in meningioma (**Supplementary Figures 2A, B**). The numbers of CD4 and CD163+ cells tended to be higher in recurrent meningiomas than in primary meningiomas (P = 0.057 and 0.084, respectively) (**Supplementary Figure 2C**).

## Discussion

Although meningiomas are typically benign intracranial tumors, the recurrence rates of WHO grade I, II, and III meningiomas have been reported as 7.2%, 29.6%, and 72%, respectively (25, 26). Therefore, prognostic factors are essential for personalized postoperative therapeutic interventions. The prognostic factors reported most frequently were the EOR and WHO grade (3). However, preoperative prognostic factors have not been fully established.

NLR has been reported to be a useful prognostic factor for brain tumors, such as gliomas and brain metastases (**Supplementary Table 3**) (27–40). However, analyses of the prognostic significance of NLR in benign brain tumors have been limited (41–43). For meningiomas, Liang et al. demonstrated that high leukocyte count and low LMR were independent predictive factors of high-grade meningiomas (13). In our study, we also investigated the preoperative NLR, LMR, and PLR in patients with meningioma. In multivariate analysis, NLR ≥ 2.6 remained an independent prognostic factor for shorter PFS. According to literature review, the median cut-off value of NLR is 4 (range: 2.5–7), which is relatively higher than the cut-off value in our study (**Supplementary Table 3**). This may be associated with the difference between benign and malignant tumors. Further analyses with benign tumors are needed.

Although a substantial number of WHO grade I meningiomas recur (24, 25), few prognostic factors have been established (4). In our study, a subgroup analysis demonstrated that NLR ≥ 2.6 was also significantly associated with shorter PFS in patients with WHO grade I meningiomas. Ki-67 LI has been reported to be useful in predicting meningioma recurrence (6, 7) and is frequently used in a clinical setting. However, Roser et al. reported that there was no statistically significant correlation between Ki-67 LI and recurrence-free survival in patients with WHO grade I meningioma (8), which was compatible with our results. Therefore, NLR may be a novel prognostic factor for WHO grade I meningiomas in addition to all grades of meningiomas. NLR can be obtained with preoperative laboratory tests, which allows us to select patients who require adjuvant therapy.

The reason why higher NLR is associated with poor prognosis remains unclear because few studies have been conducted to evaluate the relationship between peripheral blood and the tumor microenvironment (29, 44). Tumor-infiltrating lymphocytes and tumor-associated macrophages have been reported to be associated with the poor prognosis of meningioma (45, 46). Our study also revealed that the numbers of CD4+ lymphocytes and CD163+ macrophages tended to be higher in recurrent meningiomas than in primary tumors. However, we did not find a significant correlation between peripheral NLR and infiltration by these inflammatory cells. In addition, a previous study reported that NLR does not necessarily correlate with WHO grade (14). Further analysis is needed to confirm the biological role and involvement of peripheral NLR.

Besides the retrospective study design, several limitations of our study should be mentioned. First, the data were too limited to evaluate patient-reported outcomes beyond the window of overall survival because most patients were still alive. Further long-term investigative follow-up is needed. Second, we excluded factors that could affect laboratory tests, such as steroid use before laboratory tests. Third, patient backgrounds may differ from that of the reference which included only the gross total removal cases (25) because our institution performs a large number of skull base surgeries. Fourth, a rare subtype of meningioma (e.g., lymphoplasmacyte-rich meningioma) was not included in the present study. Another limitation was the paucity of the number of paired tumor tissues. A larger number of the paired samples must be studied to confirm our findings.

## Conclusion

We investigated the prognostic significance of preoperative hematological inflammatory markers in patients with meningioma. Preoperative NLR ≥ 2.6 was significantly associated with poor prognosis in WHO grade I meningiomas in addition to all grades of meningiomas. NLR can be obtained easily and cost-effectively from routine preoperative laboratory tests and thus represents a novel prognostic factor for meningiomas.

## Data Availability Statement

The original contributions presented in the study are included in the article/**Supplementary Material**. Further inquiries can be directed to the corresponding author.

## Ethics Statement

The studies involving human participants were reviewed and approved by Institutional Review Board of Keio University. The patients/participants provided their written informed consent to participate in this study.

## Author Contributions

YK and KY conceived the study design. YK, KK, and YM were responsible for data collection. NT, YK, and RT conducted histological analysis. YK and RT organized database and were responsible for statistical calculations and drafted the article. All authors contributed to the article and approved the submitted version.

## Funding

This work was supported in part by grants from the Japan Society for the Promotion of Science (JSPS) (18K19622 to MT).

## Conflict of Interest

The authors declare that the research was conducted in the absence of any commercial or financial relationships that could be construed as a potential conflict of interest.
