# Supplementary material for: Prognostic Significance of Preoperative Neutrophil-to-Lymphocyte Ratio in Patients With Meningiomas
Source: Front Oncol. 2020 Nov 24;10:592470. doi: 10.3389/fonc.2020.592470 (PMC7732694; doi:10.3389/fonc.2020.592470)
Supplement: Supplementary file 1 [file Table_1.docx]

Supplementary Material

**Supplementary Table 1.** Histological subtypes of meningiomas

| **WHO grade** | **All cases** | **Histology** | **No.** |
| --- | --- | --- | --- |
| WHO grade Ⅰ | 144 | meningothelial | 67 |
|  |  | fibrous | 20 |
|  |  | transitional | 32 |
|  |  | psammomatous | 5 |
|  |  | angiomatous | 10 |
|  |  | microcystic | 4 |
|  |  | secretory | 3 |
|  |  | lymphoplasmacyte-rich | 0 |
|  |  | metaplastic | 3 |
| WHO grade ⅠⅠ | 14 | atypical | 13 |
|  |  | chordoid | 1 |
|  |  | clear cell | 0 |
| WHO grade ⅠⅠⅠ | 2 | anaplastic | 2 |
|  |  | papillary | 0 |
|  |  | rhabdoid | 0 |

Abbreviations: WHO, World Health Organization
