# Supplementary material for: Prognostic Significance of Preoperative Neutrophil-to-Lymphocyte Ratio in Patients With Meningiomas
Source: Front Oncol. 2020 Nov 24;10:592470. doi: 10.3389/fonc.2020.592470 (PMC7732694; doi:10.3389/fonc.2020.592470)
Supplement: Supplementary file 2 [file Table_2.docx]

Supplementary Material

**Supplementary Table 2.** Receiver Operator Characteristics Analysis of NLR by Each Subgroup

| Subgroup | AUC | Optimal cut-off value | Sensitivity | Specificity |
| --- | --- | --- | --- | --- |
| Tumor status |  |  |  |  |
| Primary | 0.57 | 2.6 | 35.48 | 81.37 |
| Recurrence | 0.55 | 2.1 | 46.15 | 71.43 |
| WHO grade |  |  |  |  |
| Grade I | 0.62 | 2.6 | 40.63 | 82.14 |
| Grade II and III | 0.64 | 2.1 | 83.33 | 50.00 |
| Extent of removal |  |  |  |  |
| GTR | 0.58 | 2.6 | 44.00 | 81.52 |
| STR | 0.53 | 1.6 | 78.95 | 37.50 |
| Tumor location |  |  |  |  |
| Skull base | 0.57 | 2.6 | 29.63 | 87.65 |
| Non-skull base | 0.51 | 3.2 | 29.41 | 82.86 |
| Peritumoral brain edema (PTBE) |  |  |  |  |
| With PTBE | 0.49 | 2.9 | 30.77 | 80.43 |
| Without PTBE | 0.62 | 2.5 | 38.89 | 82.86 |

**Abbreviations:** AUC, area under the curve; WHO, World Health Organization; GTR, gross total removal; STR, subtotal removal; PTBE, peritumoral brain edema
