# Supplementary material for: Prognostic Significance of Preoperative Neutrophil-to-Lymphocyte Ratio in Patients With Meningiomas
Source: Front Oncol. 2020 Nov 24;10:592470. doi: 10.3389/fonc.2020.592470 (PMC7732694; doi:10.3389/fonc.2020.592470)
Supplement: Supplementary file 3 [file Table_3.docx]

Supplementary Material

**Supplementary Table 3.** Summary of the literature analyzing the association between preoperative NLR and OS in brain tumors

| **First author** | **Year of  publication** | **Histology** | **Sample  size** | **Cut-off  value** | **Outcomes** |
| --- | --- | --- | --- | --- | --- |
| Bambury^27^ | 2013 | glioblastoma multiforme | 84 | 4 | OS |
| McNamara^28^ | 2014 | glioblastoma | 107 | 4 | OS (from second surgery) |
| Han^29^ | 2015 | glioblastoma | 152 | 4 | OS |
| Auezova^30^ | 2016 | glioma (WHO grade I–IV) | 178 | 4 | OS |
| Kaya^31^ | 2017 | glioblastoma | 90 | 5 | OS |
| Wang^32^ | 2017 | glioblastoma | 166 | 4 | OS |
| Mitsuya^40^ | 2017 | brain metastasis | 105 | 5 | OS |
| Lopes^33^ | 2018 | glioblastoma multiforme | 117* | 7 | OS |
| Wang^34^ | 2018 | glioma (WHO grade I–IV) | 112 | 4 | OS |
| Weng^35^ | 2018 | glioblastoma | 105 | 4 | OS |
| Bao^36^ | 2018 | glioma (WHO grade I–IV) | 219 | 2.5 | OS |
| Gan^37^ | 2019 | glioma (WHO grade III and IV) | 135 | 3 | OS |
| Lv^39^ | 2019 | glioblastoma | 192 | 2.7 | OS |

Abbreviations: WHO, World Health Organization; OS, overall survival

* Stupp-protocol treated patients
