# Supplementary material for: Prognostic Significance of Preoperative Neutrophil-to-Lymphocyte Ratio in Patients With Meningiomas
Source: Front Oncol. 2020 Nov 24;10:592470. doi: 10.3389/fonc.2020.592470 (PMC7732694; doi:10.3389/fonc.2020.592470)
Supplement: Supplementary file 4 [file Image_1.pdf]

Supplementary Material

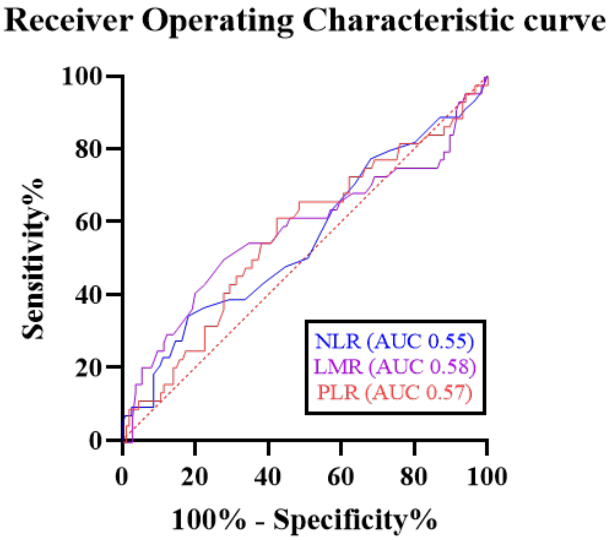

Supplementary Figure 1. Receiver operating characteristic curves

Receiver operating characteristic curves for hematological inflammatory markers, including neutrophil-to-lymphocyte ratio (NLR), lymphocyte-to-monocyte ratio (LMR), and platelet-to-lymphocyte ratio (PLR), are shown.
