# Supplementary material for: Prognostic Significance of Preoperative Neutrophil-to-Lymphocyte Ratio in Patients With Meningiomas
Source: Front Oncol. 2020 Nov 24;10:592470. doi: 10.3389/fonc.2020.592470 (PMC7732694; doi:10.3389/fonc.2020.592470)
Supplement: Supplementary file 5 [file Image_2.pdf]

## Supplementary Material

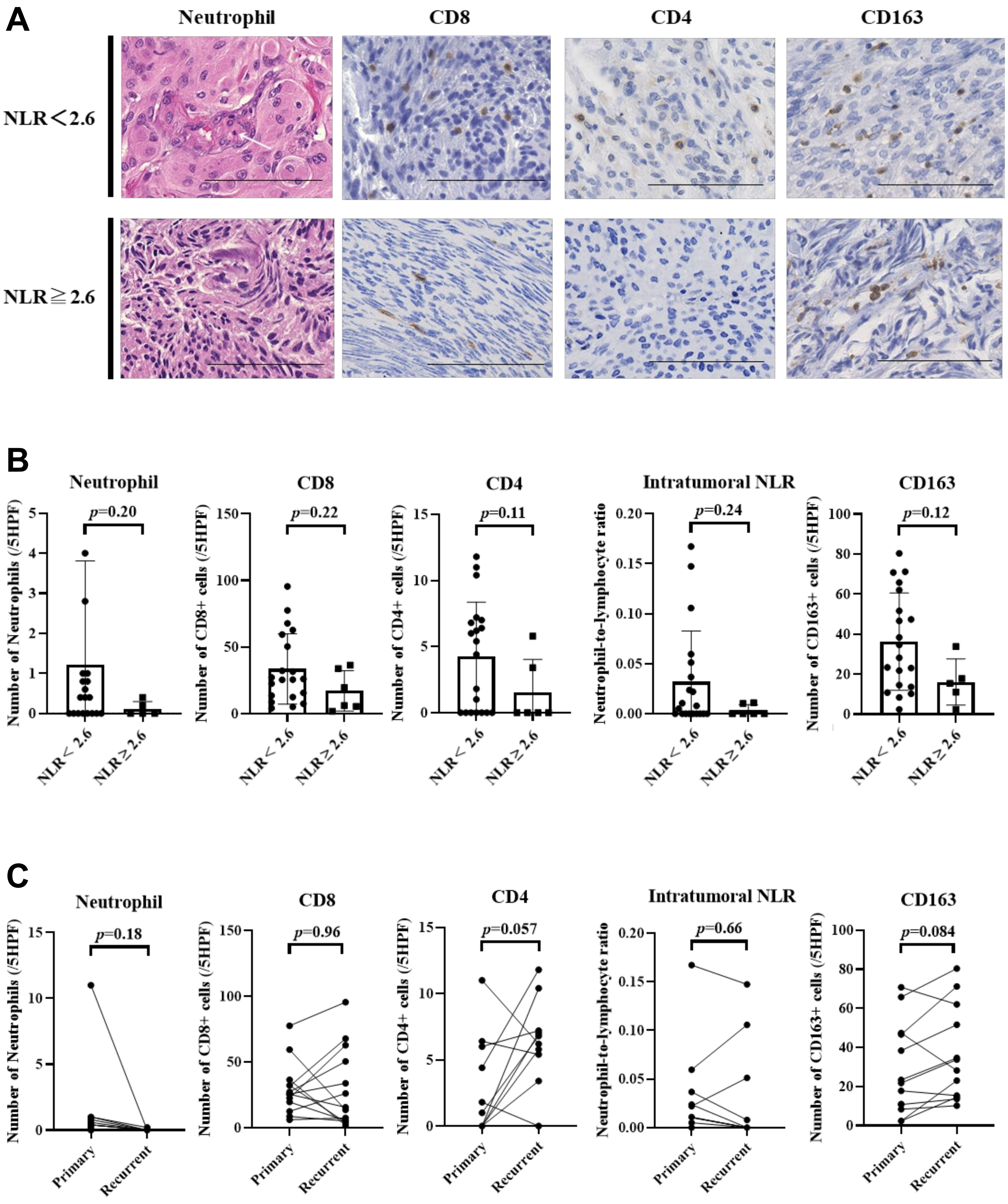

**Supplementary Figure 2. Histopathological analysis**

(A) Representative histopathological images of intratumoral neutrophils and CD8, CD4, and CD163+ cells in meningiomas with preoperative neutrophil-to-lymphocyte ratios < 2.6 and ≥ 2.6. (B) The relationship between peripheral NLR and intratumoral neutrophils and neutrophil-to-lymphocyte ratio, and CD8, CD4, and CD163+ cells. (C) The number of intratumoral neutrophils and CD8, CD4, and CD163+ cells, and intratumoral neutrophil-to-lymphocyte ratio in paired primary and recurrent meningiomas.
